# Experiences of dementia and attitude towards prevention: a qualitative study among older adults participating in a prevention trial

**DOI:** 10.1186/s12877-020-1493-4

**Published:** 2020-03-12

**Authors:** Anna Rosenberg, Nicola Coley, Alexandra Soulier, Jenni Kulmala, Hilkka Soininen, Sandrine Andrieu, Miia Kivipelto, Mariagnese Barbera, for the MIND-AD and HATICE groups, Miia Kivipelto, Shireen Sindi, Alina Solomon, Sandrine Andrieu, Nicola Coley, Hilkka Soininen, Anna Rosenberg, Edo Richard, Tessa van Middelaar, Tobias Hartmann, Carol Brayne, Edo Richard, Pim van Gool, Eric Moll van Charante, Cathrien Beishuizen, Susan Jongstra, Tessa van Middelaar, Lennard van Wanrooij, Marieke Hoevenaar-Blom, Hilkka Soininen, Tiia Ngandu, Mariagnese Barbera, Miia Kivipelto, Francesca Mangialasche, Sandrine Andrieu, Nicola Coley, Juliette Guillemont, Yannick Meiller, Bram van de Groep, Carol Brayne

**Affiliations:** 1 Department of Neurology, Institute of Clinical Medicine, University of Eastern Finland, Kuopio, Finland; 2 LEASP, UMR 1027, INSERM/Université Toulouse III Paul Sabatier, University of Toulouse, Toulouse, France; 3 Department of Epidemiology and Public Health, Toulouse University Hospital, Toulouse, France; 4 Public Health Promotion Unit, Finnish Institute for Health and Welfare, Helsinki, Finland; 5 Division of Clinical Geriatrics, Center for Alzheimer Research, Department of Neurobiology, Care Sciences and Society, Karolinska Institutet, Stockholm, Sweden; 6 School of Health Care and Social Work, Seinäjoki University of Applied Sciences, Seinäjoki, Finland; 7 Neurocenter Finland, Neurology, Kuopio University Hospital, Kuopio, Finland; 8 Institute of Public Health and Clinical Nutrition, University of Eastern Finland, Kuopio, Finland; 9 Stockholms Sjukhem, Research & Development Unit, Stockholm, Sweden

**Keywords:** Older adults, Cognitive impairment, Alzheimer’s disease, Dementia, Healthy ageing, Prevention, Risk reduction, Randomised controlled trial, Qualitative research, Family history

## Abstract

**Background:**

A better insight into older adults’ understanding of and attitude towards cognitive disorders and their prevention, as well as expectations and reasons for participation in prevention trials, would help design, conduct, and implement effective preventive interventions. This qualitative study aimed at exploring the knowledge and perceptions of cognitive disorders and their prevention among participants in a prevention trial.

**Methods:**

Semi-structured interviews were conducted among the participants of a multinational randomised controlled trial testing the efficacy of a lifestyle-based eHealth intervention in preventing cardiovascular disease or cognitive decline in community dwellers aged 65+. Participants were probed on their reasons for participation in the trial and their views on general health, cardiovascular disease, ageing, and prevention. The subset of data focusing on cognitive disorders (15 interviewees; all in Finland) was considered for this study. Data were analysed using content analysis.

**Results:**

Participants’ knowledge of the cause and risk factors of cognitive disorders and prevention was limited and superficial, and a need for up-to-date, reliable, and practical information and advice was expressed. Cognitive disorders evoked fear and concern, and feelings of hopelessness and misery were frequently expressed, indicating a stigma. Strong heredity of cognitive disorders was a commonly held belief, and opinions on the possibility of prevention were doubtful, particularly in relation to primary prevention. Family history and/or indirect experiences of cognitive disorders was a recurrent theme and it showed to be linked to both the knowledge of and feelings associated with cognitive disorders, as well as attitude towards prevention. Indirect experiences were linked to increased awareness and knowledge, but also uncertainty about risk factors and possibility of prevention. Distinct fear and concerns, particularly over one’s own cognition/risk, and high motivation towards engaging in prevention and participating in a prevention trial were also identified in connection to this theme.

**Conclusions:**

Family history and/or indirect experiences of cognitive disorders were linked to sensitivity and receptiveness to brain health and prevention potential. Our findings may be helpful in addressing older adults’ expectations in future prevention trials to improve recruitment, maximise adherence, and facilitate the successful implementation of interventions.

## Background

Cognitive impairment and dementia are a global public health priority [1], and dementia prevention or risk reduction through lifestyle management has gained increasing attention [2]. So far, a few large, long-term randomised controlled trials (RCTs) targeting multiple risk factors simultaneously have been conducted among at-risk older adults, and first results are promising [3–6]. Several other large multidomain prevention trials have been launched, or are currently planned, in diverse settings worldwide [7]. With the rise in Internet use, eHealth tools have the potential to deliver such interventions to large populations in a cost-effective manner.

The optimal design and conduct of multidomain prevention trials, which would maximise adherence and engagement in prevention during and after the trial, are however unclear. Better understanding of older adults’ dementia literacy could be valuable in this regard. Previous research has highlighted the need for increased awareness of brain health, as the general knowledge of dementia is inadequate [8, 9] and it is surrounded by a stigma [10–12]. According to the World Alzheimer Report 2019, approximately 62% of healthcare professionals and 70% of general public consider dementia a part of normal ageing, and 25% believe that nothing can be done to prevent it [12]. Importantly, lack of knowledge could hamper engagement in prevention [13]. Further insight into older adults’ attitude towards prevention, as well as expectations and reasons for participating in prevention trials, could potentially improve the design and recruitment of future interventions, facilitate their successful and sustainable implementation, and in turn, inform public health policy.

ACCEPT-HATICE [14] is a sub-study of the “Healthy Ageing Through Internet Counselling in the Elderly” (HATICE) RCT (ISRCTN48151589) which aimed at testing the efficacy of a novel eHealth tool in improving self-management of cardiovascular risk factors (CVRF) and preventing cardiovascular disease (CVD) and cognitive decline [15, 16]. Based on the ACCEPT study [17], ACCEPT-HATICE was one of the first studies to investigate at-risk older adults’ reasons for participating in a large, longer-term multidomain lifestyle prevention trial, using both quantitative and qualitative methods. Motivations were compared and investigated across the countries involved in HATICE (Finland, France, the Netherlands). In Finland, aspects related to cognitive impairment and dementia emerged in the interviews as important reasons for participation [14]. The present study, a secondary analysis focusing on the Finnish interviews, aimed at exploring in depth participants’ knowledge and perception of cognitive impairment and dementia, as well as attitude towards prevention.

## Methods

### Study population and setting

ACCEPT-HATICE study has been described in detail previously [14]. Briefly, both quantitative and qualitative approaches (questionnaires and interviews) were used to explore participants’ reasons for enrolling in HATICE, an 18-month eHealth RCT in Finland, France, and the Netherlands investigating the efficacy of a lifestyle intervention, delivered through an Internet platform, in supporting CVRF self-management and preventing CVD and cognitive decline [15, 16]. In HATICE, 2724 cognitively healthy community dwellers aged 65+, with at least two CVRFs and/or diagnosed CVD or diabetes, were randomised 1:1 to the intervention or control group. The intervention group had access to a personalised, interactive Internet platform where participants received information on CVD and CVRF prevention, set goals for lifestyle changes, and communicated with a coach who provided advice and motivational support. The control platform contained only basic information and no interactive features [18].

In the ACCEPT-HATICE study, individuals in Finland, France, and the Netherlands who met the trial eligibility criteria based on pre-screening were invited to complete an online questionnaire about their reasons to participate, preferably before the screening visit, but in some cases between screening and randomisation or shortly after randomisation. A convenience sample of respondents who agreed to be re-contacted were invited for an interview during the first three months of follow-up (on average seven weeks) after the baseline visit. In total, 341 participants completed the questionnaire (191 in Finland, 103 in France, 47 in the Netherlands) and 46 participants were interviewed (15 in Finland, 13 in France, 18 in the Netherlands) [14]. The HATICE trial and the ACCEPT-HATICE sub-study received ethical approval from the local ethics committees and all participants provided written informed consent.

The present study used qualitative data collected in the ACCEPT-HATICE study and focused on a subset of data related to cognitive impairment and dementia, a topic of discussion that was not included in the original topic list but emerged freely from the participants only in Finland [14]. For this reason, the topic could not be probed and data were not available in the French and Dutch interviews. The total number of interviews conducted for ACCEPT-HATICE in each country was pre-defined due to expected data saturation (approximately 15 per country). In Finland, 21 participants were invited by telephone and 15 individuals agreed to be interviewed. The six individuals who were not interviewed were either not reached (*N* = 3) or they refused to participate (*N* = 3). Reasons for refusal included being busy or out of town. The study population for the present analysis included all Finnish interviewees (*N* = 15), as they all spontaneously raised the topic of cognitive impairment and dementia (to which the interviewer and participants colloquially referred to as cognitive disorders). Participants in Finland were interviewed, on average, three weeks (range 1–5 weeks) after the baseline visits.

### Data collection

In June–July 2016, semi-structured face-to-face interviews with 15 participants (one interview per participant) were conducted at the University of Eastern Finland Brain Research Unit by a researcher with qualitative research experience (A.R.), using the pre-defined topic list prepared for the ACCEPT-HATICE study [14]. Topics included introduction, views on general health, CVD, ageing, and prevention, and reasons for participation in HATICE. Questions were open-ended and participants were encouraged to freely develop the discussion, while A.R. kept the conversation in-topic and ensured that topics were sufficiently covered [19]. Participants who spontaneously raised the topic of cognitive disorders during the interview (e.g. mentioned any aspects related to such conditions as a reason for participation) were probed on their perception of cognitive disorders and prevention. Research questions were defined a priori based on previous findings [20]. Examples of questions asked during the interviews are shown in Table 1. At the end of each interview, A.R. summarised verbally the conversation, giving participants the opportunity to add information or clarify their views. Interviews lasted approximately one hour each and were audio-recorded. The Consolidated criteria for reporting qualitative research (COREQ) checklist [21] is included for detailed information about methodology (Supplementary Table 1, Additional file 1).

**Table 1:** Examples of questions asked during the interviews (grouped per research question)

Knowledge of cognitive disorders and their prevention	
What do you think about cognitive disorders? What do you know about them? What do you know about the risk factors of cognitive disorders? What do you know about prevention of cognitive disorders? What do you know about the link between CVD and cognitive disorders? Do you think there is a connection between CVD and cognitive disorders?	
Perception of and feelings associated with cognitive disorders	
What kind of thoughts do cognitive disorders evoke? Why? What scares/worries you about cognitive disorders? How does it make you feel (when participants described their experiences with people affected by cognitive disorders)?	
Attitude towards prevention	
Do you believe cognitive disorders can be prevented (why/why not)? If yes, how? Is there anything one can do to reduce the risk of cognitive disorders? What motivates you towards prevention of cognitive disorders? Why did you decide to participate in the trial? What kind of expectations do you have for this trial? What kind of benefit, if any, are you expecting to get? What kind of information are you hoping to get? What did you find particularly interesting in this trial? Can you describe how cognitive disorders motivated you towards participating in this trial?	

### Data analysis

Content analysis was applied to the interview data [22]. Interviews were transcribed verbatim and excerpts related to cognitive disorders were extracted and collated. Since one of the two coders (M.B.) is not a Finnish native speaker, transcripts were translated in English by A.R. and verified by two native Finnish and fluently English-speaking colleagues. To gain an in-depth understanding of the data, coders (A.R. and M.B.) examined the transcripts independently through repeated readings and performed inductive coding without a pre-defined coding frame. A.R. performed the initial coding in Finnish; the rest of the analysis was performed in English. Transcript excerpts were divided into condensed meaning units, i.e., shortened phrases capturing the meanings of the quotes, and labelled with codes. After the initial coding, A.R. and M.B. discussed and compared their codes. Based on differences and similarities, codes were grouped into sub-categories, which were further sorted and abstracted into general categories and linked to research questions. Coders discussed and revised the sub-categories and general categories until consensus was reached. Examples of condensed meaning units, codes, sub-categories, and general categories are shown in Table 2.

**Table 2:** Examples of condensed meaning units, codes, sub-categories, and general categories

Condensed meaning unit	Code	Sub-category	General category	Research question
I'm aware of my risk factors because of family history of the diseases	Awareness of risk factors (experiences with relatives)			
Mother started using AD medications quite late, but I would like to get the medications as soon as possible	Will to get early treatment (experiences with relatives)			
My mother's AD would have progressed faster without the medications	Faster progression without medications (experiences with relatives)	Increased awareness and knowledge due to family history and/or indirect experiences		
I'm familiar with CVD and cognitive disorders because my father has both diseases	Familiar with cognitive disorders (experiences with relatives)			
I'm sure family history plays a role, my mother-in-law's mother and all my aunts had AD	Family history plays a role (experiences with relatives)		Knowledge and beliefs linked to and obtained through family history and/or indirect experiences	Knowledge of cognitive disorders and prevention
My mother-in-law's deterioration started when her husband died and she was depressed	Difficult situation in life/depression is a risk factor (experiences with relatives)			
One parent had AD and the other had CVD, so I don't know if the risk factors are same or different	Not knowing if CVD and cognitive disorders share the same risk factors (experiences with relatives)			
My brother had a healthy diet, he exercised, was slim and didn't smoke or drink, but still got AD	Getting AD despite of having healthy lifestyle (experiences with relatives)	Uncertainty about the cause and risk factors due to family history and/or indirect experiences		
I don't know what caused AD in my aunts as they all had different situations in life	Not knowing the cause (experiences with relatives)			

## Results

Interviewee characteristics are presented in Table 3. Median age was 67 years (range 66–71 years), 67% (10/15) were women, and 60% (9/15) had university level education. The proportion of participants randomised to the intervention and control group was balanced. Supplementary Table 2 in Additional file 1 summarises the demographics of the 15 Finnish interviewees in the present study, the Finnish ACCEPT-HATICE and HATICE participants, and the whole HATICE study population.

**Table 3:** Interviewee characteristics

Participant No.	University education	Employment status	Living with a partner	Randomisation
1	No	Retired	Yes	Intervention
2	Yes	Retired	Yes	Intervention
3	Yes	Retired	Yes	Control
4	No	Retired	Yes	Control
5	Yes	Retired	Yes	Intervention
6	No	Retired	Yes	Control
7	Yes	Retired	Yes	Intervention
8	Yes	Retired	Yes	Control
9	No	Retired	Yes	Control
10	No	Retired	No	Control
11	No	Retired	Yes	Intervention
12	Yes	Retired	Yes	Intervention
13	Yes	Retired	Yes	Control
14	Yes	Working part-time	Yes	Intervention
15	Yes	Working full-time	Yes	Control

For each of the pre-defined research questions, 1) Knowledge of cognitive disorders and prevention; 2) Feelings associated with cognitive disorders; and 3) Attitude towards prevention, general categories were generated to describe our findings (Table 4). Research questions and general categories, illustrated by quotes, are described in the following sections.

**Table 4:** Research questions and general categories

Knowledge of cognitive disorders and prevention	
1. Misconceptions about cognitive disorders 2. Partial knowledge of risk factors and prevention 3. Importance of early diagnosis and treatment 4. Need for up-to-date, reliable, and practical information 5. Knowledge and beliefs linked to and obtained through family history and/or indirect experiences of cognitive disorders	
Feelings associated with cognitive disorders	
1. Fear and concern 2. Stigma of cognitive disorders 3. Reasons for fear and stigma 4. Feelings linked to family history and/or indirect experiences of cognitive disorders	
Attitude towards prevention of cognitive disorders	
1. Proactive attitude 2. Motivating factors towards prevention 3. Attitude towards prevention linked to family history and/or indirect experiences of cognitive disorders 4. Motivation to participate in a prevention trial linked to family history and/or indirect experiences of cognitive disorders	

### Knowledge of cognitive disorders and prevention

To explore participants’ knowledge of cognitive disorders and prevention, five general categories were identified: 1) Misconceptions about cognitive disorders; 2) Partial knowledge of risk factors and prevention; 3) Importance of early diagnosis and treatment; 4) Need for up-to-date, reliable, and practical information; 5) Knowledge and beliefs linked to and obtained through family history and/or indirect experiences of cognitive disorders.

When probed on their knowledge of cognitive disorders, some participants admitted knowing hardly anything about the topic, and cognitive disorders were commonly described as “mysterious” (participant 15) or “hard to figure out” (participant 13). The most common misconceptions were related to the aetiology of cognitive disorders. First, there was confusion about the difference between age-related and pathological cognitive decline: several participants perceived cognitive disorders as a normal, inevitable part of ageing when diagnosed at an old age.

*“If we live long enough, we will all get it in one way or another. ( …*) *I guess it’s part of normal ageing and development.” (Participant 14).*

Second, their development was commonly attributed to genetic factors and family history and they were considered largely hereditary conditions.

*“One cannot influence the diseases which are inherited and genetic …*” *(Interviewer: Can you name any?) “Dementia.” (Participant 6).*

Despite these erroneous beliefs, many participants had acquired some knowledge of modifiable risk factors and prevention e.g. through media. Yet, this knowledge seemed partial and superficial. Depression, stressful life events, and social isolation/loneliness were mentioned as potential risk factors by some, but the role of lifestyle, except for harmful alcohol use, was described vaguely.

*“I don’t really know if there is any link [between CVD and cognitive disorders]. ( …*) *I don’t know if the risk factors are the same or different.” (Participant 1).*

*“There’s a link [between lifestyle and cognitive disorders], e.g. alcohol dementia is 100% caused by alcohol. But as far as the other [lifestyle-related] factors are concerned, I’m not sure what role they have.” (Participant 10).*

A few participants mentioned not knowing if or how cognitive disorders can be prevented, but some were able to name certain protective factors like cognitive training, computer use, hobbies like crossword puzzles, and maintaining an active social life. Physical factors, such as exercise, healthy diet, and treating hypertension, were rarely mentioned.

*(Interviewer: How do you think cognitive disorders could be prevented?) “I don’t have a clue. I don’t even know if they can be prevented.” (Participant 8)*

*“Maybe it can be slowed a little by exercising and training memory … By keeping the brain active.” (Participant 6).*

Many were doubtful or hesitant on whether and to what extent prevention is possible. Some contradicted themselves, as they first claimed that cognitive disorders can be prevented but later expressed a sense of hopelessness and resignation to fate.

*“I’ve read a lot about prevention; solve crossword puzzles, do this and do that, and you will supposedly prevent dementia. I’m sceptical about that.” (Participant 13).*

When talking about the possibility of prevention, participants often referred to secondary prevention: although the possibility to postpone disease onset and slow down its progression was acknowledged, little confidence was expressed in preventing it from occurring altogether.

*“I’m sure it [having social interaction] can prevent or at least slow it [dementia]. I guess not completely, it will come if it’s meant to be, but at least the process can be slowed.” (Participant 10).*

Despite having limited knowledge, many participants were aware of the early symptoms and different disease stages, particularly the long pre-dementia stage before the onset of severe symptoms. Consequently, they were well informed about the importance of early diagnosis and treatment for better prognosis. The role of medications was endorsed in delaying dementia onset.

*“Someone defended a PhD thesis about a blood test to detect Alzheimer’s disease (AD) even before the first symptoms, but it is not routinely used. I’ve asked many times if I could have it. As soon as it [AD] is about to start, I would like to start using medications so that it could be slowed.” (Participant 1).*

Many recognised the gaps in their knowledge and hoped to obtain up-to-date, evidence-based information about cognitive disorders from a reliable source, e.g. through participation in HATICE. Topics of interest included e.g. causes of cognitive disorders, early symptoms and clinical manifestation, and risk factors. Importantly, a need for concrete, practical, and understandable advice on prevention was expressed.

*“I was discussing with my friends if my Internet use [as a cognitively stimulating activity] will help me avoid dementia. [I would like to get] information about that. And which factors influence the development of dementia. Is there anything one can do, or not. Or is it genetic. I’m a bit torn.” (Participant 13).*

*“Does it for example say somewhere that one should solve one crossword puzzle per day or something … [Expectation is] to get concrete tips and advice on what I should do [to prevent].” (Participant 15).*

Participants’ knowledge and beliefs appeared to be obtained through family history and/or indirect experiences of cognitive disorders. Indirect experiences were linked to greater awareness and increased, yet incomplete knowledge. Participants reporting family history and/or indirect experiences named more risk and protective factors and mentioned more often the importance of early diagnosis and treatment to slow down the disease progression. However, they tended to emphasise the role of genetics.

*“Our mother started the medication quite late ( …*). *I don’t know if it’s possible to detect it [the disease] myself but at least when others notice that there’s something wrong... It would be possible to intervene earlier. To get the medication early. ( …*) *God knows how fast the [my mother’s] disease would have progressed without it.” (Participant 7).*

*“I’m sure family history plays a role. ( …*) *For example, my mother-in-law and her mother and all my grandmother’s sisters had AD. ( …*) *I was once told that I have a 50% chance to get it.” (Participant 10).*

Despite increased knowledge, some participants disclosing family history seemed uncertain about the potential risk factors because they were not able to find any common denominator between the affected individuals in their family.

*“My aunts definitely didn’t have an unhealthy lifestyle. ( …*) *And my mother-in-law was very healthy. But a difficult situation in life can affect the outbreak of a cognitive disorder. [My mother-in-law’s decline] started when her husband died. ( …*) *But some of my aunts were spinsters, some were married, and some were widowed … So, I don’t know what caused it. That they all got AD.” (Participant 10).*

### Feelings associated with cognitive disorders

To explore participants’ perceptions of and feelings associated with cognitive disorders, four general categories were identified: 1) Fear and concern; 2) Stigma of cognitive disorders; 3) Reasons for fear and stigma; and 4) Feelings linked to family history and/or indirect experiences of cognitive disorders.

When probed on the topic of cognitive disorders, participants expressed general fear and concern over such conditions. In addition, they frequently mentioned having specific concerns over their own cognitive status or risk of cognitive disorders.

*“[A reason to participate was] to have a memory check-up. It would be interesting to know if I have problems already. At least my short-term memory has got significantly worse.” (Participant 5).*

Cognitive disorders appeared to evoke more concern than mere ageing or other diseases, including CVD or cancer. The thought of getting a cognitive disorder at a young age, namely at their age or earlier, seemed particularly disturbing.

*“Now that I was diagnosed with breast cancer, I hope I won’t start acting like my mother-in-law. Thinking that even the smallest ailments are symptoms of cancer or something. If I have cancer and it spreads, so be it. The only disease that concerns me is dementia, my mother has dementia. Some everyday situations make me think that I already have symptoms.” (Participant 13).*

*“It’s awful if memory deteriorates, especially at a young age.” (Participant 12).*

Participants also expressed feelings of hopelessness, misery, and despair. Cognitive disorders were commonly perceived as terrifying conditions, mainly due to their irreversible, progressive nature and lack of treatments. Thus, cognitive disorders seemed to be surrounded by a stigma.

*“Cognitive disorders make me sad and they evoke fear because if you get it, that’s it. There is no going back. It’s sad to think about it when you’re still healthy.” (Participant 7).*

*“My mother has AD, that’s what I fear the most. It’s such a dreadful disease that even a sudden death would be better.” (Participant 1).*

Fear and stigma were related to the deterioration of cognitive functions and personality changes which interfere with the ability to interact with others. Furthermore, loss of functional abilities and independence evoked concerns, as they subject an individual to a situation where he/she is forced to rely on others for help.

*“( …*) *One needs the help of others and is dependent on them because one doesn’t remember. Others could take advantage of it.” (Participant 12).*

Cognitive disorders were also frequently described as a burden not only for the persons themselves, but also for others, particularly the next of kin.

*“My mother doesn’t feel scared, she is alright because she doesn’t remember anything. But it’s sad for me to watch her.” (Participant 7).*

The level of distress ranged from a simple concern to great anxiety elaborated on in detail. Feelings seemed to relate to indirect experiences of cognitive disorders, as fear and worry were mostly expressed by participants who disclosed family history of cognitive disorders. Of the 15 interviewees, 11 spontaneously reported having family history and/or indirect experiences and 10 of them mentioned being highly concerned.

*“My siblings had diabetes and AD and they died. My sister’s husband also had AD. Of course it concerns me.” (Participant 2).*

Witnessing the cognitive and functional deterioration of close relatives/friends and watching them go through the end-stages of the disease when constant care is needed evoked distinct fear and anxiety. Because of such experiences, some participants appeared reluctant to learn about their personal disease risk if such information was available.

*“I know how hard the final stages are for the family. Our grandmother didn’t recognise anyone else but me, and when she saw her reflection in the mirror, she asked who this stranger was. ( …*) *Then she became physically unable to function. Dirtied places with her feces ( …*). *I wouldn’t wish that for myself.” (Participant 10).*

*“I would not want to find out yet if I will get AD. ( …*) *It’s hard to live with that information, especially after witnessing the last stages of my brother and sister.” (Participant 2).*

Family history and/or indirect experiences of cognitive disorders were also linked to how participants perceived and monitored their own health and cognition. Many participants who disclosed family history and/or indirect experiences mentioned being worried about their memory and reported subjective cognitive decline over time.

*“Now that her [my mother’s] memory is gone, I’ve started to notice that my memory has deteriorated.” (Participant 8).*

Participants described how a family member’s cognitive disorder makes one conscious of his/her own memory, and even if one is generally not easily worried about health, even minor forgetfulness in everyday life might feel alarming (see previous quote by participant 13).

### Attitude towards prevention of cognitive disorders

To explore participants’ attitude towards prevention of cognitive disorders, four general categories were identified: 1) Proactive attitude; 2) Motivating factors towards prevention; 3) Attitude towards prevention linked to family history and/or indirect experiences of cognitive disorders; and 4) Motivation to participate in a prevention trial linked to family history and/or indirect experiences of cognitive disorders.

In line with the fact that the protective role of cognitively stimulating activities was widely acknowledged among the participants, some had adopted a proactive attitude towards prevention and reported having already started to solve crossword puzzles, read books, and play memory games prior to enrolment in HATICE.

*“That’s why I’ve started to play skruuvi [a card game], like a memory game. I think it can be useful in that regard [prevention].” (Participant 14).*

Fear and family history of cognitive disorders were mentioned as key motivating factors towards lifestyle changes and engagement in prevention.

*(Interviewer: What motivates you towards prevention?) “Fear, having seen in my parents how severe these diseases are.” (Participant 1)*

Like the knowledge and feelings, attitude towards prevention was linked to family history and/or indirect experiences of cognitive disorders. Some participants who disclosed family history did not believe in the beneficial effects of healthy lifestyle based on their personal experiences. Some were uncertain, again due to the discrepancy between their experiences and what they had heard about prevention.

*“My brother was a picture of health and had a healthy diet for his whole life. No alcohol, cigarettes or anything. He was slim, worked hard, exercised, and still he got diabetes and AD. ( …*) *I guess there’s nothing one can do about it, there’s nothing my brother could have done.” (Participant 2).*

In addition to prevention as such, family history and/or indirect experiences of cognitive disorders were linked to motivation to participate in a prevention trial. Participants who talked about their experiences with affected people often mentioned it as a reason for being interested in HATICE and cognitive disorders.

*(Interviewer: What reasons did you have for participation?) “I’m interested in cognitive disorders; they might run in my family and my mother had a cognitive disorder.” (Participant 12)*

Furthermore, those with family history of cognitive disorders wanted to enrol in HATICE to gain access to detailed information about their health status. Interest in learning their personal disease risk through cognitive and genetic assessments and blood tests was frequently expressed. Such assessments were thought to facilitate a reliable prediction of disease risk and potential early detection of cognitive disorders.

*“I thought that since they run genetic tests I will find out if I carry dementia genes. But apparently that’s not the case.” (Participant 3).*

*“My father has both CVD and cognitive disorder. Of course, I’m interested in my current status. I get something out of it [participation] myself, memory tests and blood tests.” (Participant 9).*

## Discussion

This study involved cognitively healthy older adults enrolled in a lifestyle prevention trial and showed that family history and/or indirect experiences of cognitive disorders were linked to knowledge of and feelings associated with such conditions, as well as attitude towards prevention and willingness to participate in a prevention trial.

Although an increased dementia literacy is expected in highly educated trial participants [23], we found that the knowledge of cognitive disorders and their genetic and lifestyle-related risk factors was generally scant and superficial. Our findings are consistent with population-based surveys indicating that knowledge of cognitive disorders is limited even among educated older adults in high-income countries, and there is a misconception that cognitive impairment and dementia are a part of normal ageing and not preventable [8, 9, 12]. Social and cognitive activities, whose role was shown by previous studies to be better recognised than that of e.g. exercise or CVRF management [24–26], were commonly perceived as relevant for prevention also in our study population. This included also eHealth tools, such as brain trainers and other online applications. Awareness of the most effective means to prevent cognitive disorders through social engagement and cognitive training could still be superficial. Overall, consistent with the ACCEPT-HATICE study [14], participants of the present study expressed a need for practical and understandable information about prevention. Despite having some superficial knowledge about risk and protective factors, participants rarely seemed to be able to translate the meaning of this research information at a personal level. In future trials, up-to-date scientific evidence on risk factors and prevention should not only be incorporated into the content of the interventions, but also communicated in a pragmatic way, which would allow participants to understand how one can affect his/her own dementia risk. Available dementia risk scores and tools [27, 28] could be useful in this regard. Importantly, considering that lack of knowledge could be perceived as a barrier towards behavioural and lifestyle change for dementia prevention [13] and knowledgeable individuals may be more likely to pursue an active and healthy lifestyle [29], promoting awareness among older adults should also be considered a priority for public health policies.

Consistent with our findings suggesting that knowledge was linked to family history and/or indirect experiences of cognitive disorders, previous studies reported that a personal relationship with a person affected by a cognitive disorder might be associated with better understanding of modifiable risk factors, as well as the beneficial effects of healthy diet, avoiding stress, and engaging in social, mental, and physical activities [9, 25, 26]. In our study, however, despite claiming to believe in prevention, those who spoke about their indirect experiences of cognitive disorders tended to attribute a decisive role to genetic factors. Hopelessness and a deterministic view that cognitive disorders can be slowed but not prevented completely was generally expressed. This type of contradiction and irrationality on one hand reflects the need for reliable and understandable evidence-based information; on the other, it demonstrates how family history of a disease and real-life experiences with affected individuals might shape a person’s perception of the disease. This has been observed in previous qualitative studies in the context of cognitive disorders [30] and other diseases, e.g. genetic conditions like haemophilia [31]. While family history of cognitive disorders could motivate towards engaging in prevention, it could also act as a barrier if the risk reduction potential is considered minimal due to high chance of heredity. Given that healthy lifestyle or lifestyle changes lower dementia risk and have beneficial effects on cognition even among individuals with genetic susceptibility for dementia [32, 33], identifying and addressing such beliefs and doubts when encouraging older adults to improve lifestyle and inviting them to prevention trials would be important to facilitate engagement and adherence.

In line with previous findings [13, 26], family history and indirect experiences of cognitive disorders were in our study linked to fear and worry, particularly over one’s own risk and cognitive status. This facilitated participation in HATICE and engagement in prevention, also in the everyday life outside the trial context. Our findings are supported by a study suggesting that individuals with experiences of, or concerns over, cognitive disorders have a positive attitude towards undertaking preventive actions and confidence in personal risk reduction [29]. Studies on other chronic diseases (diabetes, CVD) showed that family history of a disorder might be linked to perceived threat [34]. According to the Health Belief Model, a concept described and used in prior literature [35], this perceived threat influences motivation towards prevention and behaviour change. Family history and perceived threat, reflecting the combination of perceived personal risk of a disorder and its perceived severity, could increase interest in prevention and diagnostic testing [36] but also cause anxiety and decrease motivation [36, 37]. With regard to cognitive disorders, previous qualitative research demonstrated that fear and stigma could motivate older adults towards prevention [13] but also make them passive – and even prevent from seeking help when worried (Akenine et al., unpublished observations). In the ACCEPT-HATICE study, individuals worried about their health named medical monitoring as an important reason for participation in HATICE, as they felt the need to get examined for reassurance but perceived access to healthcare often as limited [14]. Collectively, these results and the present findings suggest that family history and/or indirect experiences of cognitive disorders may motivate some older adults to seek medical advice and information about their health status and disease risk in a trial.

Previous studies reported that a perceived high risk of AD and family history of cognitive disorders might motivate individuals towards enrolling in hypothetical AD drug trials [38, 39] and increase willingness to undergo genetic or diagnostic assessments [26, 40–43]. Lawrence and colleagues observed that diagnostic confirmation was an important incentive for participation among cognitively impaired older adults [40]. Findings are nevertheless inconsistent [44, 45]. Although a perceived high risk of AD was associated with increased interest in receiving information about one’s genetic and/or diagnostic status [46], the opposite was true for individuals with self-reported cognitive complaints or family history of AD [46] and former caregivers of AD patients [40]. In our study, similar reservations were expressed by some participants who reported family history and/or indirect experiences of cognitive disorders. In future preventive interventions targeting older adults, the possibility to receive additional medical monitoring to complement regular healthcare could be emphasised to facilitate recruitment. Nevertheless, possible unrealistic expectations regarding genetic or diagnostic assessments should be addressed and managed.

### Strengths and limitations

Through our qualitative approach, we had the opportunity to individually probe several older adults on specific topics related to cognitive disorders and gain an in-depth understanding of their views on attitude towards prevention. Double coding by two independent researchers and the iterative analysis process strengthened the analysis. Use of COREQ checklist [21] enabled a rigorous conduct of the study. Although the analysis was performed in English rather than in the original language, translation was checked by two colleagues fluent in both languages, and it is unlikely that such procedure altered the results. Potential selection bias, small sample size, and low heterogeneity of the study population are the main limitations of this study. ACCEPT-HATICE participants were overall younger, more educated, and had a slightly more favourable CVD risk profile than the other HATICE participants, and were therefore not representative of general older population [14]. Including more participants could potentially have led to additional insights and alternative conclusions; however, saturation was deemed to be achieved. It is noteworthy that participants were interviewed after baseline, as engaging in the intervention could have affected their perceptions. However, we consider this unlikely because interviews were conducted only 1–5 weeks after randomisation. In fact, when probed on their experiences of the trial and the HATICE platform, the majority had not yet logged in nor set goals for lifestyle changes. Also, the results did not appear to differ by randomisation group.

Finally, the fact that the topic of cognitive disorders was not included in the pre-defined topic list of the ACCEPT-HATICE study, but probed only when spontaneously raised by the interviewees, can be considered a limitation. Because of this, the topic could not be investigated starting from a more general conceptual framework and including data from all three countries involved in HATICE, like in the main ACCEPT-HATICE study. Different HATICE recruitment strategies in each country and differences in culture and healthcare settings [14, 16] may explain why the topic of cognitive disorders was raised only by the Finnish interviewees. Conducting this sub-study in all three countries could have potentially strengthened our results and improved their applicability in other geographical, cultural, or healthcare settings.

## Conclusions

Our findings indicate that family history and/or indirect experiences of cognitive disorders might be linked to older adults’ knowledge and perceptions of cognitive disorders and prevention, as well as to motivations to participate in a prevention trial. Due to concerns over their own health and risk, older adults with indirect experiences of cognitive disorders may be particularly responsive to issues related to brain health and the potential of prevention. Our findings may inform and facilitate the design of future prevention trials, particularly as regards the recruitment and selection of suitable populations and information offered as part of the interventions. Furthermore, our findings may be helpful in successfully addressing and managing participants’ expectations in future trials, potentially leading to increased adherence. Finally, considering that the identification of knowledge gaps and health beliefs in a given target population is a prerequisite for successful health education, our findings may have implications for public health policy and implementation of prevention programmes (e.g. design and content of public health awareness campaigns).

## Supplementary information

**Additional file 1: Table S1.** COREQ checklist **Table S2.** Demographics of the Finnish interviewees, all Finnish ACCEPT-HATICE participants, Finnish HATICE participants, and all HATICE participants.

## Data Availability

In order to guarantee confidentiality and anonymity of the participants, transcripts and coding analysed in the current study cannot be made public. Individual applications for data are considered. For more information, please contact Mariagnese Barbera, mariagnese.barbera@uef.fi.

## Abbreviations

ACCEPT-HATICE: Mixed-method sub-study of the Healthy Ageing Through Internet Counselling in the Elderly (HATICE) trial
AD: Alzheimer’s disease
COREQ: Consolidated criteria for reporting qualitative research
CVD: Cardiovascular disease
CVRF: Cardiovascular risk factor
HATICE: Healthy Ageing Through Internet Counselling in the Elderly
RCT: Randomised controlled trial
