# Supplementary material for: Experiences of dementia and attitude towards prevention: a qualitative study among older adults participating in a prevention trial
Source: BMC Geriatr. 2020 Mar 12;20:99. doi: 10.1186/s12877-020-1493-4 (PMC7068959; doi:10.1186/s12877-020-1493-4)
Supplement: Supplementary file 1 — Additional file 1: Table S1. COREQ checklist Table S2. Demographics of the Finnish interviewees, all Finnish ACCEPT-HATICE participants, Finnish HATICE participants, and all HATICE participants. [file 12877_2020_1493_MOESM1_ESM.docx]

**Supplementary Table 1.** COREQ checklist

| **No.** | **Item** | **Description** | **Described in text (section)** |
| --- | --- | --- | --- |
| **Domain 1**: Research team and reflexivity | | | |
| 1 | Interviewer | Rosenberg A | Methods,  Data collection |
| 2 | Researchers’ credentials (involved in data analysis) | Rosenberg A: MSc,  Barbera M: PhD | No |
| 3 | Researchers’ occupation (involved in data analysis) | Rosenberg A: early-stage researcher, Barbera M: postdoctoral researcher | No |
| 4 | Gender | All researchers are female | No |
| 5 | Experience and training | See body of text | Methods,  Data collection |
| 6 | Relationship established | No previous relationship established with the participants | No |
| 7 | Participants’ knowledge of the interviewer | Participants knew the professional background  of the interviewer | No |
| 8 | Interviewer characteristics | Rosenberg A conducts research on prevention of dementia | No |
| **Domain 2**: Study design | | | |
| 9 | Methodological  information and theory | See body of text | Methods,  Data analysis |
| 10 | Sampling | See body of text | Methods,  Study population and setting |
| 11 | Method of approach | See body of text | Methods,  Study population and setting |
| 12 | Sample size | See body of text | Methods,  Study population and setting |
| 13 | Non-participation | See body of text | Methods,  Study population and setting |
| 14 | Setting of data collection | See body of text | Methods,  Data collection |
| 15 | Presence of non-participants | No others were present |  |
| 16 | Description of sample | See body of text, Table 3 & Supplementary Table 2 in Additional file 1 | Results; Table 3, Supplementary Table 2 in Additional file 1 |
| 17 | Interview guide | See body of text and Table 1 | Methods,  Data collection; Table 1 |
| 18 | Repeat interviews | See body of text | Methods,  Data collection |
| 19 | Audio/visual recording | See body of text | Methods,  Data collection |
| 20 | Field notes | Not performed |  |
| 21 | Duration | See body of text | Methods,  Data collection |
| 22 | Data saturation | See body of text | Methods,  Study population and setting |
| 23 | Transcripts returned | Not performed |  |
| **Domain 3**: Analysis and findings | | | |
| 24 | Number of data coders | See body of text | Methods,  Data analysis |
| 25 | Description of coding tree | See examples in Table 2 | Table 2 |
| 26 | Derivation of themes | See body of text | Methods,  Data analysis |
| 27 | Software | Not used |  |
| 28 | Participant checking | Not performed |  |
| 29 | Quotations presented | See body of text | Results |
| 30 | Consistency between  data and findings | See body of text | Results |
| 31 | Clarity of major themes | See body of text and Table 4 | Results;  Table 4 |
| 32 | Clarity of minor themes | See body of text. Not all minor themes were not discussed  due to word limits. | Results |

**Supplementary Table 2.** Demographics of the Finnish interviewees, all Finnish ACCEPT-HATICE participants, Finnish HATICE participants, and all HATICE participants

| Characteristics | Finnish  ACCEPT-HATICE interviewees (N=15) | Finnish  ACCEPT-HATICE participants (N=191) | Finnish  HATICE participants (N=885) | All  HATICE participants (N=2724) |
| --- | --- | --- | --- | --- |
| Age, years | 67 (66–69) | 68 (67–70) | 69 (67–70) | 69 (67–73) |
| Female | 10 (66.7%) | 107 (56.0%) | 502 (56.7%) | 1297 (47.6%) |
| University education | 9 (60.0%) | 99 (51.8%) | 454 (51.3%) | 1120 (41.1%) |
| Retired | 13 (86.7%) | 159 (83.1%) | 793 (89.6%) | 2286 (83.9%) |
| Living with a partner | 14 (93.3%) | 155 (81.2%) | 694 (78.4%) | 1999 (73.4%) |

Data are median (interquartile range) or N (%).
